# Portable Low-Cost Electronic Nose Based on Surface Acoustic Wave Sensors for the Detection of BTX Vapors in Air

**DOI:** 10.3390/s19245406

**Published:** 2019-12-08

**Authors:** Daniel Matatagui, Fabio Andrés Bahos, Isabel Gràcia, María del Carmen Horrillo

**Affiliations:** 1 SENSAVAN, Instituto de Tecnologías Físicas y de la Información (ITEFI), CSIC, Serrano 144, 28006 Madrid, Spain; carmen.horrillo.guemes@csic.es; 2 Instituto de Ciencias Aplicadas y Tecnología (ICAT), Universidad Nacional Autónoma de México, Ciudad Universitaria, Ciudad de México 04510, Mexico; fbahos@gmail.com; 3 Departamento de Electrónica y Telecomunicaciones, Tecnoparque, Centro de la Industria, la Empresa y los Servicios, SENA, Neiva 410004, Colombia; 4 Instituto de Microelectrónica de Barcelona (IMB), CSIC, Campus UAB, 08193 Bellaterra, Spain; isabel.gracia@imb-cnm.csic.es

**Keywords:** portable electronic nose, Love-SAW sensor, surface acoustic wave, Fe_2_O_3_ sensitive layer, gas detection

## Abstract

A portable electronic nose based on surface acoustic wave (SAW) sensors is proposed in this work to detect toxic chemicals, which have a great potential to threaten the surrounding natural environment or adversely affect the health of people. We want to emphasize that ferrite nanoparticles, decorated (Au, Pt, Pd) and undecorated, have been used as sensitive coatings for the first time in these types of sensors. Furthermore, the proposed electronic nose incorporates signal conditioning and acquisition and transmission modules. The electronic nose was tested to low concentrations of benzene, toluene, and xylene, exhibiting excellent performance in terms of sensitivity, selectivity, and response time, indicating its potential as a monitoring system that can contribute to the detection of toxic compounds.

## 1. Introduction

There is a great need to improve the current monitoring and control of hazardous gases in the quality of air, mainly in cities, with the principal goal of protecting the health of citizens. The Directive 2008/50/EEC on “Ambient Air Quality and Cleaner Air for Europe” was adopted by the European Parliament and the European Council to inform the general public about this subject.

At present, pollutant samples are usually collected in the field for further analysis in the laboratory through conventional techniques [1,2,3,4,5,6], such as gas chromatography/mass spectrometry, UV spectroscopy, colorimetric, high-performance liquid chromatography, etc., but these systems present a major inconvenience in terms of inefficient time use due to many factors, such as sample transportation, analyte desorption, pre-concentration, and data transmission. Additionally, these systems are expensive and require specialists to operate them, and in general, they cannot measure in situ due to their large size and weight. Therefore, the development of fast, portable, and low-cost technologies that can upload measurements in real time for air pollution analysis is a great challenge. These new technologies could replace the monitoring stations located in areas where citizens and workers are exposed to high levels of these toxic gases. 

Electronic nose technology satisfies these requirements. Many types of electronic noses, mainly resistive ones, have been developed to monitor, discriminate, and classify a range of volatile organic compounds (VOCs) for very different fields of application (environment, feeding, security, and health) [7,8,9,10,11]. However, surface acoustic wave (SAW) electronic noses are not common in the literature, as existing systems are neither compact nor portable, even though these sensors are very sensitive and able to work at room temperature [12,13,14,15].

Because of this, in the present research, our interest has been to develop a portable and wireless electronic nose (analyzer), where the sensor array is formed by different SAW sensors, designed to monitor a group of aromatic hydrocarbons in the air, such as benzene, toluene, and xylene (BTX), which are gases that are harmful to health; benzene because of its carcinogenic nature, and toluene and xylene due to their dangerous effects on the central nervous system [1]. 

The Occupational Safety and Health Administration (OSHA) defines the Short-Term Exposure Limit (STEL)—the concentration of a substance in air which may not be exceeded over a 15 min period—as 5 ppm for benzene, 150 ppm for toluene, and 150 ppm for xylene.

As far as we have seen, this is the first SAW electronic nose fabricated with microelectronic and microfluidic technologies, hence its compactness, portability, and low cost. Another important characteristic of its design is the ease with which the sensor module can be exchanged, for example, between SAW, SH-SAW, Love-SAW, and others. This is, as far as we know, the first electronic nose made with this type of interchangeable sensor module. In addition, this electronic nose incorporates the signal conditioning and the acquisition and transmission modules. 

In this research, we intend to highlight the electronic nose prototype developed with Love-SAW sensors, as they present the advantages of a great surface confinement of the acoustic energy by the guide layer (which increases the sensitivity to gases) and the capacity to propagate the waves across the solid–gas interface without suffering excessive acoustic wave attenuation [16,17]. In addition, Love-SAW sensors have revealed a higher sensitivity to gases than Rayleigh SAW sensors [18].

In our first works, the performance of this type of sensors was studied for different VOCs, using mainly polymers as coating materials [19,20], although we later employed nanomaterials due to their great specific detection surface [21,22]. Also noteworthy, as a novelty in this research, is the use of ferrite nanoparticles decorated and undecorated as sensitive coatings, since they have been used for other types of sensors, above all for semiconductor sensors [23,24], but not for SAW sensors. 

Therefore, this electronic nose could replace the current complex techniques to measure these compounds and other VOCs in the air, in a fast and effective way. The advantages obtained from this type of instrumentation are very important for real-time monitoring and control of toxic VOCs, which significantly affect the development of serious human diseases. Thus, this instrument would bring great benefits to society.

## 2. Materials and Methods

### 2.1. Surface Acoustic Wave Device

**Figure 1:**
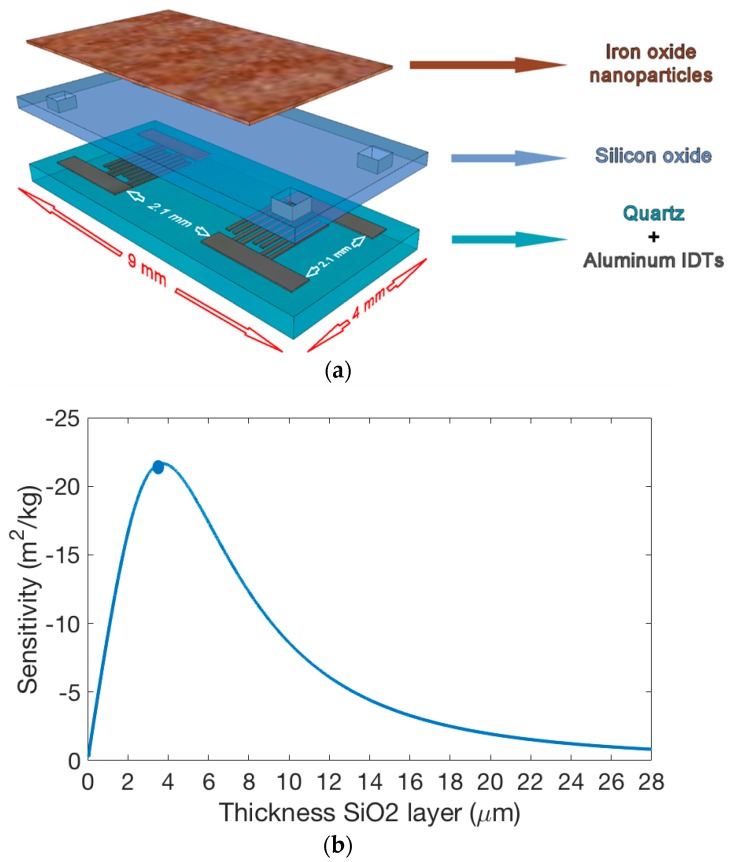
(**a**) 3D scheme representing layers (quartz substrate, interdigital transducers (IDTs), SiO_2_, and nanostructured iron oxide) of the Love-wave sensor. (**b**) Mass sensitivity for different thicknesses of the SiO_2_ guiding layer.

The SAW sensor range includes Love-wave (LW) sensors based on shear horizontal (SH) waves, guided by a layer with a lower propagation velocity than that of the piezoelectric substrate (ST-90°X quartz). The energy of the wave is confined to the guiding layer, and any perturbation in it affects the acoustic wave velocity (pressure, temperature, mass, elasticity). The LW sensors used in the present work were designed with a delay line (DL) configuration. This device is based on a micro-electromechanical system composed of a piezoelectric material with facing input/output aluminum interdigital transducers (IDTs) on its surface, working at a 28 μm wavelength (λ), with a separation between IDTs of 2100 μm (Figure 1). In previous work, the least-mean-square error fitting method was applied to the dispersion equation in order to find, from experimental data, the density and the shear modulus values for deposited SiO_2_ [25], and from the obtained results the mass sensitivity was calculated. Therefore, SH waves were guided by a 3.5 μm-thick SiO_2_ layer, obtaining the synchronous frequency of the devices around 160 MHz, with the sensitivity of the fabricated device (indicated by a blue dot) close to the maximum sensitivity at 3.7 μm (Figure 1b).

### 2.2. Electronic Nose

**Figure 2:**
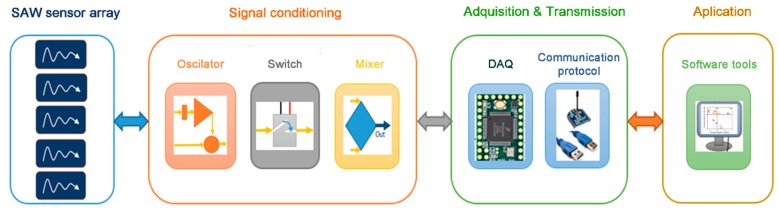
General scheme of different modules of the electronic nose: surface acoustic wave (SAW) sensor array, signal conditioning, acquisition and transmission and application software.

**Figure 3:**
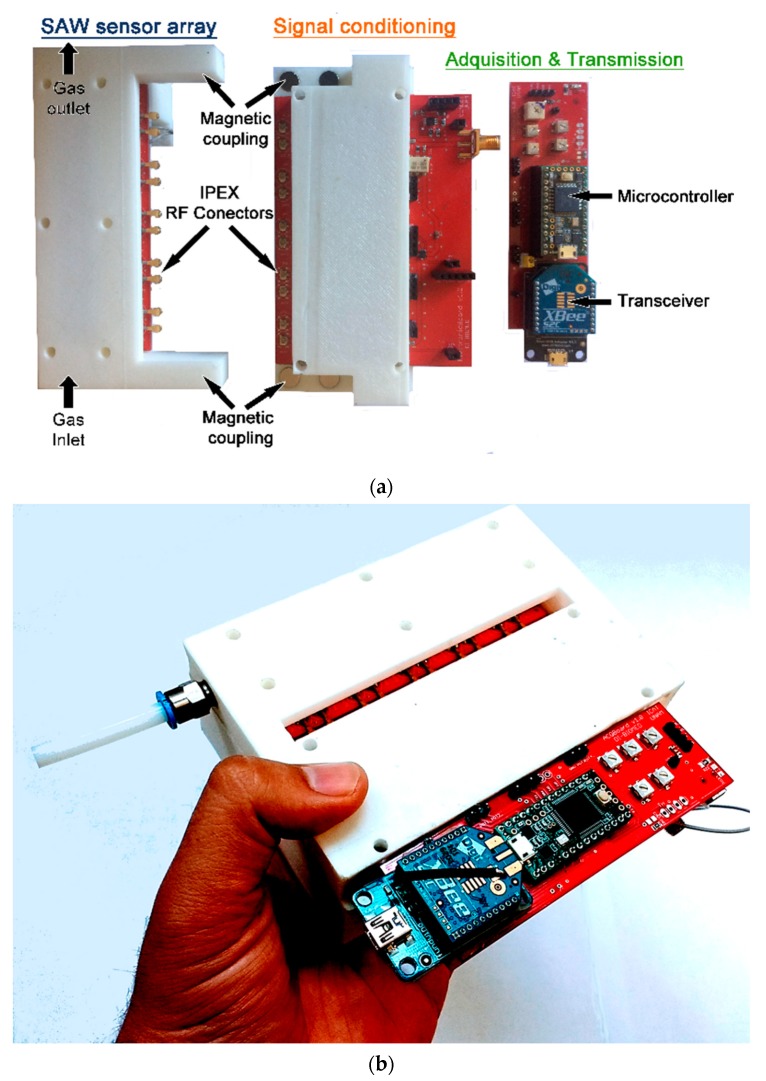
Electronic nose (**a**) with separated modules: SAW sensor array, signal conditioning, and acquisition and transmission; (**b**) with assembled modules.

The system was designed through a modular architecture, composed of four subsystems, each of them with a specific objective (sensor array, signal conditioning, data acquisition and transmission, and application software) (Figure 2). This modularity in subsystems makes it easy to use them in different applications and allows subsystem expansion without affecting the whole system (Figure 3). Electronic enclosures and the sensor array chamber were made with a 3D printer.

#### 2.2.1. Sensor Array Module

Four different sensors, plus a reference device, comprised the LW sensor array. The sensors were developed with different sensitive layers to achieve a specific fingerprint for the analytes of interest, and the reference was an LW device without a sensitive layer. The sensitive layers were based on iron oxide (Fe_2_O_3_) nanoparticles (Sigma-Aldrich 544884, Madrid, Spain). Sensor 1 (S1) was obtained from pure dispersion and the others were obtained by combining pure solution with nanoparticles of Au (Metrohm-Dropsens AUNP-COL) (sensor 2, S2), Pt (Metrohm-Dropsens PTNP-COL) (sensor 3, S3), and Pd (Metrohm-Dropsens PDNP-COL) (sensor 4, S4). In order to obtain sensitive layers from nanoparticle dispersions, the SiO_2_ guiding layer was covered with 25 µL of the sample, and then the assemblage was spun at a speed of 4000 rpm.

The LW sensors were housed in a printed circuited board (PCB) and connected through gold-plated pins for optimal transmission of the RF signal to the signal conditioning module.

#### 2.2.2. Signal Conditioning Module

The sensor array and signal conditioning modules were mechanically coupled by magnets and electrically connected by means of IPEX contacts, since they are inexpensive micro-miniature RF connectors and because the smaller coaxial cables they use are much easier to deal with. The present structure of the electronic nose allows for quick changes of the sensor array, without altering the signal conditioning and acquisition and transmission modules.

The signal conditioning module introduces each sensor device of the array into a feedback loop composed of two amplification states and a directional coupler, satisfying the criteria for oscillation: the total phase shift in the loop is 2π*n* (*n* = integer) and the gain over the closed loop is 1. The coupled output from the directional coupler was used to obtain, in real time, a sample of the frequency from the oscillator without interrupting the main power flow. The electronic nose is based on a heterodyne configuration, so a multiplexor selects one of the four sensor-oscillator signals and forwards it to a single output, which is mixed with the signal from the oscillator based on the reference LW device (established as the local oscillator), thus obtaining a new signal from the difference of the two original frequencies. Therefore, the reference LW device has a dual propose: to reduce the operating frequency and to compensate for external factors such as change of pressure, temperature, and noise, among other disturbances.

#### 2.2.3. Acquisition and Transmission Module

The signal conditioning and acquisition and transmission modules were coupled through vertical pins, and therefore the acquisition and transmission module was assembled on top of the signal conditioning module.

**Figure 4:**
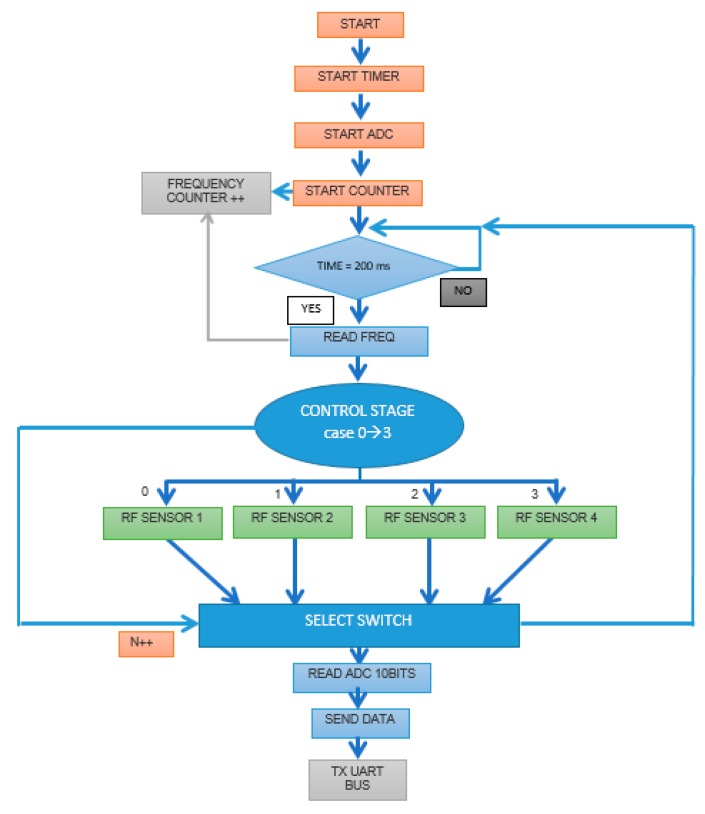
Flowchart of microcontroller firmware to control the electronic nose and its data acquisition.

The acquisition and transmission module was based on a microcontroller. The multiplexer of the signal conditioning module takes inputs from multiple oscillators and is controlled through the microcontroller’s digital outputs, which route the desired signal to the RF mixer. The mixer output signals are then amplified, filtered, and finally acquired by the microcontroller’s analog-to-digital converter (ADC) port, which is programmed as a frequency counter (Figure 4). This module incorporates hardware for wired communication through FT232R USB-UART and wireless communication with XBEE protocol. This module can enable real-time coordinates from a global positioning system (GPS). 

#### 2.2.4. Application Software

The data acquired by the microcontroller were transmitted to a PC, and an open source custom application was developed to display and store, in real time, the experimental sensor data. In addition, this application allows the electronic nose (microcontroller) to be quickly and easily reconfigured.

To statistically analyze the variability of the sensor array’s measurements, principal component analysis (PCA) was applied to the data from the sensor array’s response. PCA is a chemometric linear, unsupervised, and pattern recognition technique for reducing the number of dimensions of a numerical dataset in a multivariable problem. Mathematically, this method applies a linear transformation to the data and results in a new space of variables called principal components. The principal components are ordered, and thus the greatest variance is on the first coordinate (called first principal component, PC1), the second greatest variance is on the second coordinate, PC2, and so on. PC1 and PC2 allow the visualization of dataset main information in 2D representation. The plot of scores is normally used for studying the distribution of the data clusters.

A probabilistic neural network (PNN) was also applied. This is a type of neural network with radial basis transfer functions, which measures the distance between input vector and the training vectors. 

### 2.3. Experimental Setup

**Figure 5:**
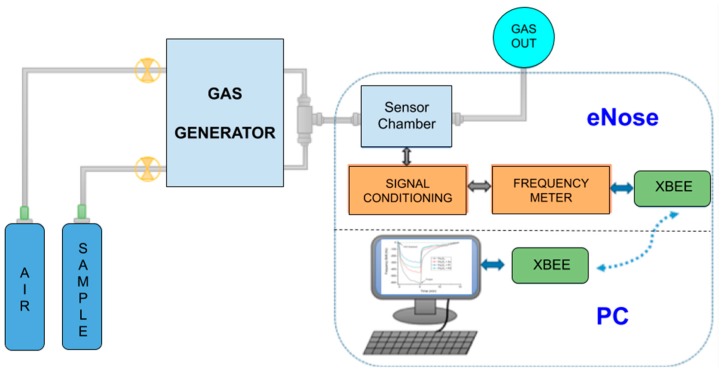
Experimental setup for the eNose characterization.

A computerized gas calibration was used to vary the benzene, toluene, and xylene concentrations in synthetic air, wherein the electronic nose was tested. These concentrations were achieved by using mass flow controllers, connected to the PC by Modbus protocol. The total constant flow of the gas was kept at 200 mL·min^−1^ and the exposure and the purge times were 5 and 10 min, respectively (Figure 5).

## 3. Results and Discussion

### 3.1. Electrical Characterization

**Figure 6:**
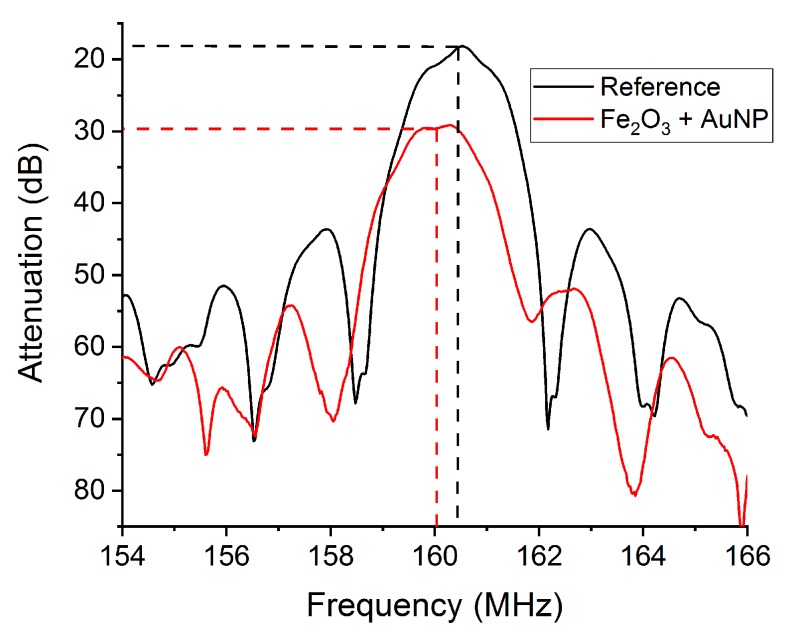
Spectral response of the LW sensor before (black) and after (red) deposition of the sensitive layer that combines iron oxide and gold nanoparticles.

The electronic nose was developed as a low-cost and adaptive system for use in different environments and applications. This was taken into account in its design and development, giving rise to the following characteristics:

- Power supply. The electronic nose was designed to be fed by a 5 V source, which allows the electronic nose to be plugged into a laptop by USB connector, or any of the range of 5 V batteries available on the market. The typical current consumption was 200 mA, requiring a nominal power of 1 W. Depending on the specific application, very small batteries can be used for a few hours of autonomy, or larger batteries (about the size of the electronic nose) can be coupled outside of the signal processing module for a few days of autonomy.
- Frequency range. Different SAW devices are supported by the electronic nose we developed, such as Rayleigh, shear horizontal, Love, multi-guiding layer Love, etc. [18,20,25]. Despite the fact that a similar design is used for SAW devices, each of them works at different frequencies, and consequently the electronic nose was designed to support frequencies from 120 MHz to 200 MHz; the low-pass filter can be modified with a cutoff frequency to up 1 GHz.
- The insertion losses of LW devices were lower than 20 dB. Due to a mechanical damping effect, significant insertion losses can be induced by the sensitive layer. This was the case with the sensitive layer based on the combination of iron oxide and Au (Figure 6), which increased the insertion losses of the LW device by 12 dB, resulting in a total attenuation of ~30 dB for sensor S2 [26]. To ensure the correct performance of the electronic nose, the signal condition module was designed to support LW devices with up to 38 dB of insertion losses.
- Frequency noise. The noise of such a sensor system is highly important to its ability to quantify the limit of detection (LOD) or the minimum detectable value, which was estimated to be three times the signal-to-noise rate (SNR). The typical noise for sensors with this electronic nose was 5 Hz/min, therefore the LOD for each sensor is the concentration of gas that induced a change in the sensor’s working frequency of 30 Hz.
- Crosstalk. When the electromagnetic communication between oscillator circuits resulted in an attenuation equal to or greater than 50 dB, this was characterized as crosstalk. This effect was considered when measuring the induced frequency shift among oscillators, which gave a result of lower than 1% in any case.
- The electronic nose parameters are displayed in Table 1.

### 3.2. Gas Characterization

To prove the performance of the electronic nose, we conducted a test with three aromatic hydrocarbons (VOCs) classified as pollutants with a significant risk to the environment and to the health of humans, such as benzene, toluene, and xylene. This group of pollutants is well known as BTX. Gases were introduced during a 5 min exposure process in the sensor array chamber at concentrations of 10, 25, and 50 ppm, and then were removed from the chamber with synthetic air for 10 min in the purge process. Each exposure-purge period was repeated three times.

**Figure 7:**
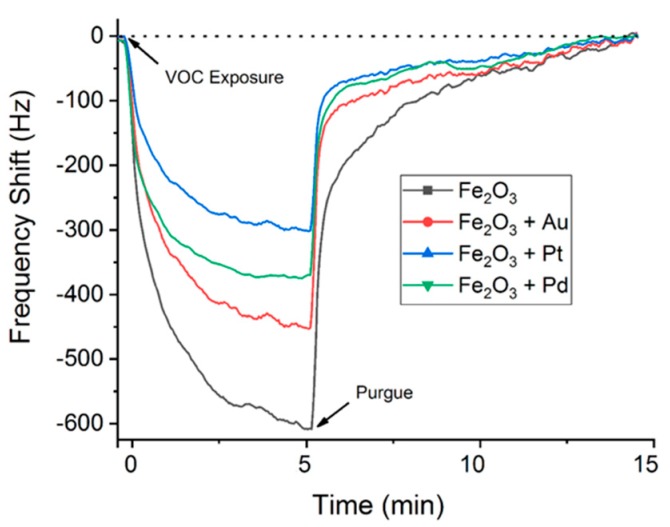
Real-time response of the SAW array of the electronic nose to 50 ppm of xylene.

Figure 7 illustrates the real-time response of the array’s sensors to 50 ppm of xylene. All sensors showed a notable response between 300 Hz (sensor with sensitive layer composed of iron oxide and Pt nanoparticles, S1) and 600 Hz (sensor with pure iron oxide nanoparticles, S3). In these cases, the LOD of the sensors for a typical frequency noise is 1.25 ppm of xylene for S1 and 2.5 ppm for S3. The response time is an important parameter for gas sensors in practical applications, and it can be determined by defining *τ_90_* as the time taken to reach 90% of the frequency shift. As shown in Figure 6, the response times of sensors were 2.55, 2.45, 2.62, and 2.05 min for S1, S2, S3, and S4, respectively, which were similar in the case of S1, S2, and S3, however S4 presented a faster response. The average response times of the sensor array for the three exposure–purge periods were obtained for 50 ppm of benzene, toluene, and xylene (Table 2).

**Figure 8:**
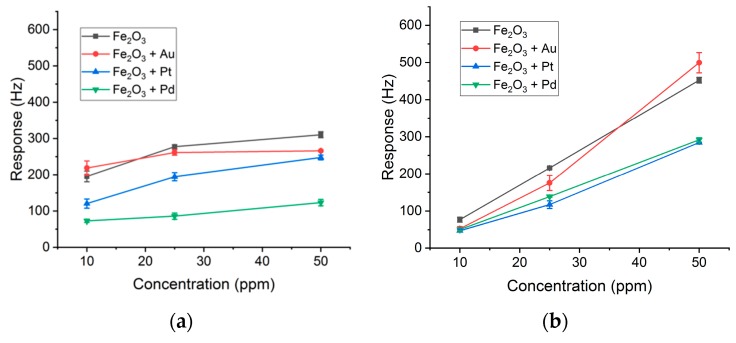

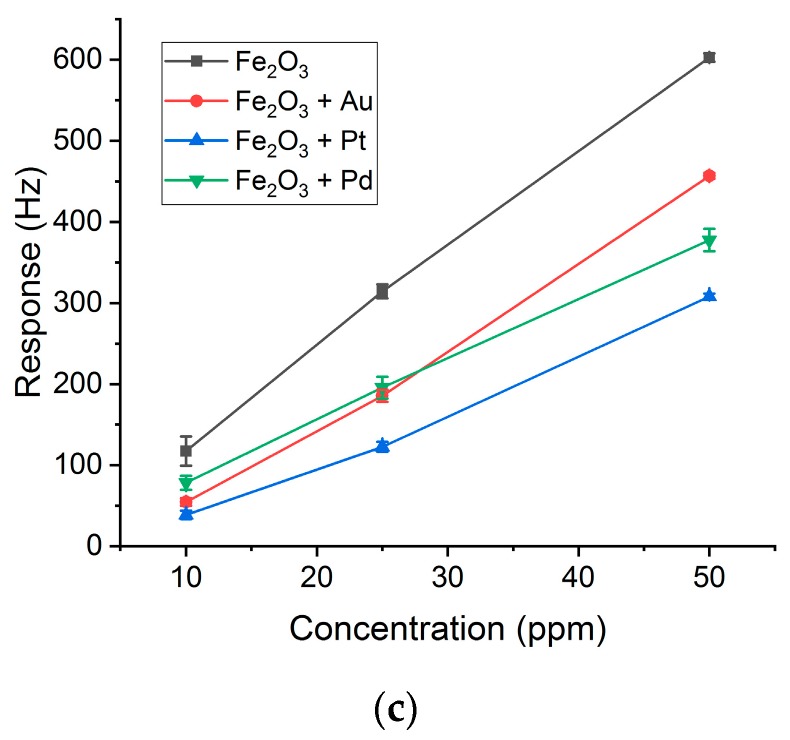
Calibration curves of the array of SAW sensors for (**a**) benzene, (**b**) toluene, and (**c**) xylene. The vertical error bars are the standard deviation of triplicated measurements for each concentration.

The frequency shifts (peaks) at the end of the exposure time were taken as responses, and averages for three measurements of each concentration were calculated to obtain calibration curves. In this context, for each BTX compound, the responses were plotted against the concentration of each tested sensor. Figure 8 shows the results obtained, and the vertical error bars represent the standard deviation of the triplicated response peaks. In all cases, responses of the sensors increased with higher concentrations. The responses of toluene and xylene present a linearity as a function of the concentration. However, for higher benzene concentrations, this linearity is lost, because the sensors are working in a saturated stage.

### 3.3. Statistical Treatment

**Figure 9:**
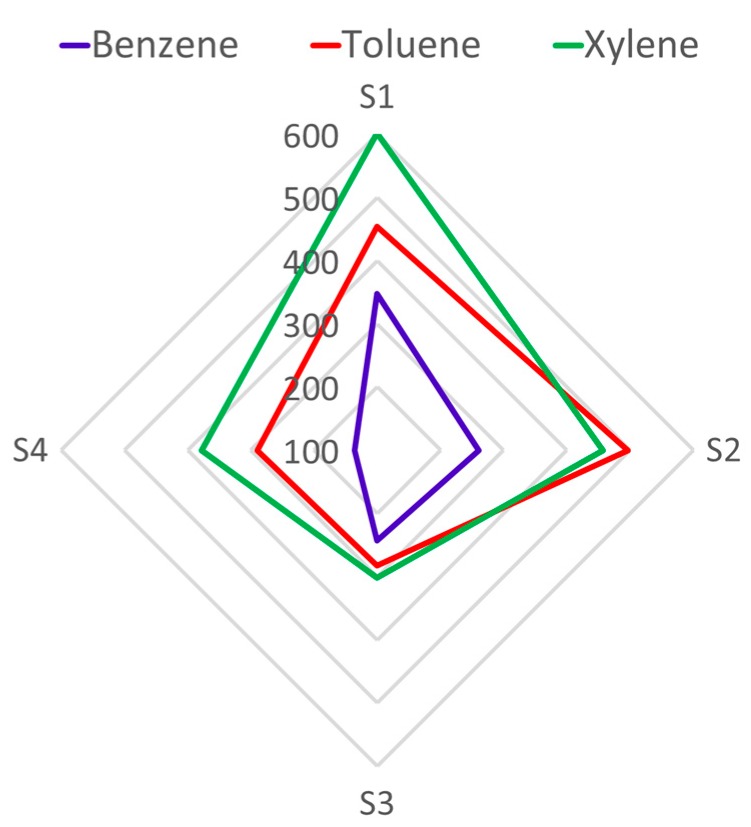
Radial representation of the sensor array’s responses to 10 ppm of benzene, toluene, and xylene.

**Figure 10:**
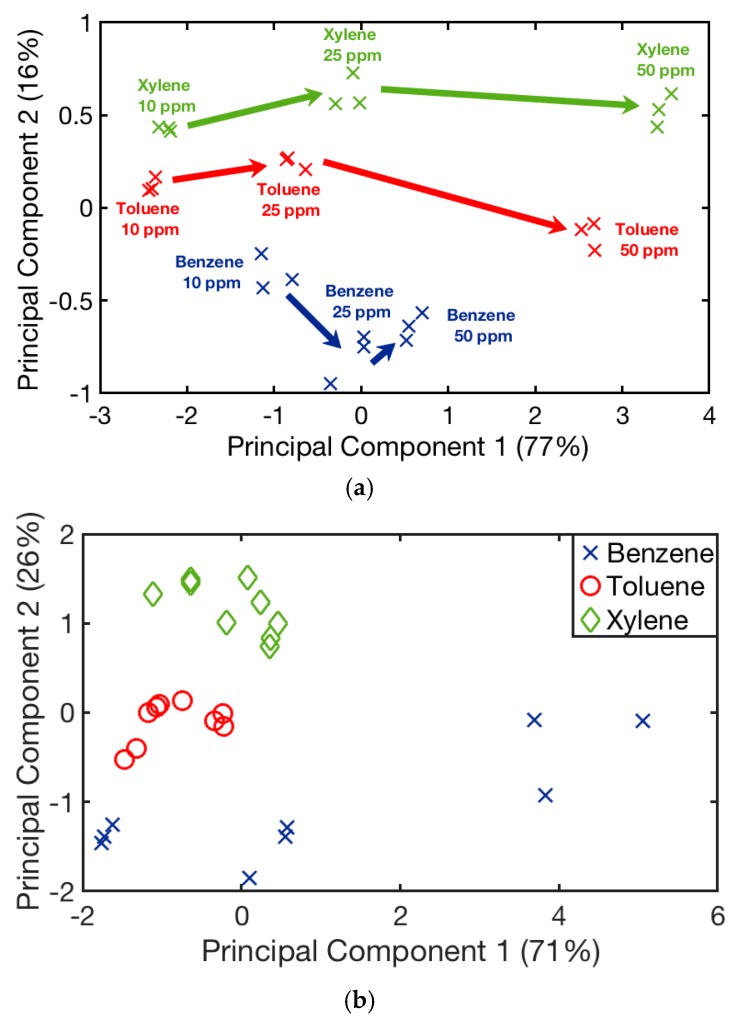
(**a**) Principal component analysis applied to data for discrimination of benzene (blue), toluene (red), and xylene (green) at different concentrations. The arrow direction indicates the increase in concentration. (**b**) Principal components analysis applied to data/concentration for discrimination of benzene (blue), toluene (red), and xylene (green).

Figure 9 shows the average responses of all sensors to 50 ppm of each target gas, with a distinct pattern for each one, allowing visual discrimination. Principal component analysis allowed us to summarize and to visualize the information in a dataset containing individual responses. The scores of the three toxic gases were plotted for the most important components—PC1 and PC2—and they are represented in Figure 10a, which was used to study the statistical discrimination. The data was clearly clustered by each BTX and concentration, and a good separation between them was achieved. In addition, the arrow direction indicates the increase in concentration for each target. The obtained results illustrate that the proposed electronic nose, combined with PCA data analysis, could recognize different VOCs. 

However, gas concentration changes tend to shadow the reaction of sensors with analytes, since the sensor response contains both qualitative (sensor–analyte interaction) and quantitative (analyte concentration) information. In order to remove the quantitative information, data have been divided by concentration before applying PCA (Figure 10b). In this case, the PCA confirmed again the presence of well-defined patterns in sensors responses, indicating a clear discrimination of the three VOCs. Toluene and xylene show clustered data; however, benzene displays a scattered data as a result of the saturation regime in which the sensor works. In addition, PC1, PC2, and PC3 have been used to train a PNN, and their performance has been evaluated with the leave-one-out validation method. This method consists of training *N* distinct nets (in this case, *N* is the number of measurements) by using the remaining vector, excluded from the training set. This procedure was repeated *N* times until all the vectors were validated, achieving in this case a 100% correct classification.

## 4. Conclusions

Over the last few decades, surface acoustic wave sensors have proved their efficiency in terms of sensitivity to chemical compounds. In the present paper, the design, development, and operation of a portable, low-cost, and wireless electronic nose has been described, showing its potential as a real-time monitoring system that can contribute to the detection of toxic chemicals.

The electronic nose was designed to work in different environments, incorporating novel, easy, and fast mechanisms for gas detection. The system was designed using modular architecture, composed of four subsystems, (sensor array, signal conditioning, data acquisition and transmission, and application software) which makes it very self-contained and versatile. For the first time in this type of sensor, sensitive coatings were based on ferrite nanoparticles, decorated (Au, Pt, Pd) and undecorated. The electronic nose has proved to detect benzene, toluene, and xylene at concentrations of 10, 25, and 50 ppm, with high sensitivity and high selectivity, showing a distinct pattern for each toxic agent, and a high efficiency to discriminate between BTX using principal component analysis.

## Figures and Tables

**Table 1:** Electronic parameters of the electronic nose.

Parameter	Minimum	Typical	Maximum	Units
Power supply	4.0	5.0	5.4	V
Current consumption	-	200	350	mA
Power	-	1000	1890	mW
Frequency range	120	160	200	MHz
Gain	30	35	40	dB
Frequency noise	2	5	10	Hz/min

**Table 2:** Average response times of the sensor array for the three exposure–purge periods.

Sensor	Benzene 50 ppm (min)	Toluene 50 ppm (min)	Xylene 50 ppm (min)
S1	3.13	1.22	2.43
S2	1.66	1.32	2.38
S3	1.48	1.27	2.48
S4	2.12	1.25	2.13
